# The Consolidated Framework for Implementation Research (CFIR): progress to date, tools and resources, and plans for the future

**DOI:** 10.1186/1748-5908-10-S1-A12

**Published:** 2015-08-20

**Authors:** Laura Damschroder, Carmen Hall, Leah Gillon, Caitlin Reardon, Caitlin Kelley, Jordan Sparks, Julie Lowery

**Affiliations:** 1 Center for Clinical Management Research, VA Ann Arbor Healthcare System, Ann Arbor, MI, USA; 2 Gusek Hall Consulting, Roseville, MN 55113, USA

## Objective

The objective of this presentation is to introduce the Consolidated Framework for Implementation Research (CFIR), present results of a literature synthesis of studies citing the CFIR, highlight improvements expected in a second version of the framework, and present tools and resources available for researchers using the CFIR that will be available on a newly revamped website.

## Methods

In a series of four interactive virtual panels, we elicited user feedback from implementation research novices and experts on needed CFIR tools. In addition, we searched multiple databases to identify articles that cited the CFIR. Articles were characterized as original research, syntheses, study protocols, or general background references.

## Results

347 published articles cited the CFIR, with an average of growth rate of four additional articles per week; fifty-one were original research, protocols, or syntheses. Recommendations were extracted from these articles and used to inform updates for CFIR V2. Refinements will include improved clarity in definitions for existing constructs, addition of new constructs, and better framing of the purpose and uses of CFIR. The CFIR website was significantly redesigned with the addition of new tools and resources including: 1) an interview guide creation tool; 2) links to a periodically updated bibliography of articles applying the CFIR; 3) two published quantitative measures related to organizational change mapped to CFIR constructs; 4) in-depth guidance on how to apply the CFIR; and 5) plans for the future. A demonstration of the publically available website will be provided along with the URL.

## Advances for D&I

The CFIR brings clarity to commonly studied constructs by suggesting clear and consistent terms and definitions that can be applied across diverse settings, within and outside healthcare. Use of the CFIR is growing. CFIR V2 along with tools, resources, and published applications, will help researchers collectively advance the science of implementation.

## Funding

Department of Veteran Affairs, Health Services Research & Development Quality Enhancement Research Initiative (QUERI) (Grant # RRP 12-494).

